# PReSaFe: A model of barriers and facilitators to patients providing feedback on experiences of safety

**DOI:** 10.1111/hex.12516

**Published:** 2016-11-16

**Authors:** Aoife De Brún, Emily Heavey, Justin Waring, Pamela Dawson, Jason Scott

**Affiliations:** ^1^ School of Nursing Midwifery and Health Systems University College Dublin Dublin Ireland; ^2^ Social Policy Research Unit University of York York UK; ^3^ Nottingham University Business School Nottingham University Nottingham UK; ^4^ Faculty of Health and Life Sciences Northumbria University Newcastle upon Tyne UK; ^5^ Institute of Health & Society Newcastle University Newcastle upon Tyne UK

**Keywords:** patient experience, patient reporting, patient safety, qualitative research

## Abstract

**Objective:**

The importance of involving patients in reporting on safety is increasingly recognized. Whilst studies have identified barriers to clinician incident reporting, few have explored barriers and facilitators to patient reporting of safety experiences. This paper explores patient perspectives on providing feedback on safety experiences.

**Design/Participants:**

Patients (n=28) were invited to take part in semi‐structured interviews when given a survey about their experiences of safety following hospital discharge. Transcripts were thematically analysed using NVivo10.

**Setting:**

Patients were recruited from four hospitals in the UK.

**Results:**

Three themes were identified as barriers and facilitators to patient involvement in providing feedback on their safety experiences. The first, *cognitive‐cultural*, found that whilst safety was a priority for most, some felt the term was not relevant to them because safety was the “default” position, and/or because safety could not be disentangled from the overall experience of care. The *structural‐procedural* theme indicated that reporting was facilitated when patients saw the process as straightforward, but that disinclination or perceived inability to provide feedback was a barrier. Finally, *learning and change* illustrated that perception of the impact of feedback could facilitate or inhibit reporting.

**Conclusions:**

When collecting patient feedback on experiences of safety, it is important to consider what may help or hinder this process, beyond the process alone. We present a staged model of prerequisite barriers and facilitators and hypothesize that each stage needs to be achieved for patients to provide feedback on safety experiences. Implications for collecting meaningful data on patients' safety experiences are considered.

## 1 Introduction

Following highly publicized failings in patient care in the UK, increased importance is placed on identifying and learning from patient safety incidents with the goal of safeguarding against future deficiencies.^1^, ^2^ One of the most commonly adopted mechanisms to identify patient safety incidents is health‐care professional incident reporting.^3^ However, there are shortcomings with this approach, including the culture of blame and resistance to excessive administrative duties^4^ which can result in the under‐reporting of patient safety incidents.^5^, ^6^ In conjunction with recent inquiries (eg *Freedom to Speak Up* 7), there are growing calls for patient involvement in safety‐reporting and learning processes. When willing and able, there is “considerable scope” for patients to play an active role in ensuring that their care is safe^8^ by providing feedback^9^ through reporting incidents and/or evaluating safety experiences.

Patient experience measures have been shown to provide meaningful information to health‐care professionals regarding experiences of safety.^8^ Patients can be involved in safety by speaking up at the point of care, making formal complaints or providing feedback via surveys.^10^ Research has also demonstrated positive associations between patient experience measures and other outcome measures, such as patient adherence, clinical processes and safety culture.^11^, ^12^ Significantly, patients can provide a different perspective on safety to health‐care staff, which can inform approaches to managing safety and risk; patients can recognize issues not seen or reported by staff^13^ and identify risks to which staff may have become desensitized. A recent review of patient reporting on safety concluded that patients can play a role as part of a larger “error detection jigsaw” to improve quality and care.^10^

However, there are many barriers to patients engaging with current reporting structures and systems.^10^ Individuals may fear being branded as “difficult” patients if they are seen as questioning staff or their quality of care^14^, ^15^ and thus may be reluctant to report safety concerns. Patients may also adopt a “self‐protection strategy” by avoiding reporting safety issues to staff who appear unresponsive, uninterested or unapproachable.^16^ Such findings underline the importance of providing explicit opportunities for patients to report safety concerns and also serve to highlight safety as a process which is contingent on, and coproduced by, the interactions and relationships between patients and health‐care practitioners.^17^, ^18^, ^19^

Through reporting safety incidents, patients could operate as an extra source of learning or intelligence,^20^ or “safety buffers,” within the health‐care system.^21^, ^22^, ^23^ Previous findings emphasize the necessity of understanding and addressing the barriers and facilitators to engaging patients in safety reporting. Identified barriers include patients' own illness severity and cognitive characteristics, the relationship between the patient and the health‐care practitioner, contextual factors and the perception of being subordinate to medical professionals.^15^

Given the particularly high‐risk process of care transfers,^23^, ^24^, ^25^, ^26^, ^27^ this study recruited patients who had been discharged from hospital to understand their perceptions and experiences of safety in the context of their discharge and care transfer. Indeed, Coulter et al.^28^ have recently identified a clear need for further research on capturing patient experiences when transitioning between organizations. The aim of this study was to examine the barriers and facilitators to patients reporting on these safety experiences.

## 2 Methods

### 2.1 Data collection

In total, 28 patients participated in the study; 10 participants were female (36%) and 18 were male (64%). The mean age of participants was 68 (range 53‐86). Patients were given an invitation letter to participate in a semi‐structured interview after completing a safety survey,^29^ which was handed out to them by health‐care staff upon discharge and completed once they had arrived at their next destination.^23^ The safety survey was codesigned with patient representatives,^29^ based on how patients perceive safety.^21^ Patient representatives were also consulted in designing the patient interview guide and contributed to the wider design and conduct of the study via an advisory group. Patients were recruited from four clinical areas (cardiac, care of older people, orthopaedics and stroke) using convenience sampling after expressing an interest in participating in an interview when returning the survey. Inclusion criteria for patients were that they were able to give informed consent, aged 18 or over and able to take part in an English language interview (one participant was interviewed with the help of an interpreter). Table 1 provides a description of the participants' survey responses and care transfers.

**Table 1:** Rich description of participant characteristics

Participant number	Gender	Age	Ethnicity
104	Male	83	English
462	Male	61	White
761	Male	80	White English
980	Female	55	White British
1189	Male	68	English
1867	Male	53	White English
2450	Male	56	White British
2494	Male	77	English
2590	Female	81	English
2593	Female	68	White English
3319	Male	86	British/English
3408	Male	80	English
3445	Female	56	British
3954	Male	82	White
4300	Male	54	White English
4679	Female	79	White British
5583	Male	59	British
5767	Female	80	White British
5853	Male	65	English
5945	Male	65	British
6227	Female	67	White British
6427	Female	54	British
6725	Female	65	White European
7701	Male	71	White British
8182	Male	62	White British
9748	Male	69	White British
11100	Female	56	White British
11597	Male	60	White British

Interview questions included a focus on barriers and enablers to provide useful feedback on their own safety within care transfers, and also included general health questions, general safety questions and questions relating to their experience of care transfers. The researchers did not define “safety” for patients; instead, we were interested in their conceptualizations and understanding of the term, as well as its perceived relevance to them. The interview schedule was refined iteratively throughout data collection. The study received favourable ethical opinion from National Health Service (NHS) Research Ethics Committee (ref: 13/YH/0372), and R&D approval was obtained from the NHS Trusts taking part in the research.

### 2.2 Data analysis

Interviews were transcribed verbatim, then coded and analysed using NVivo10 qualitative analysis software. Drawing on the approach outlined by Braun and Clarke,^30^ all transcripts were closely read and initial codes generated and recorded by one author's initials removed for review anonymization. After initial coding, codes were refined and combined into overarching themes. The themes were refined and arranged into conceptual groupings. The final codes and themes were discussed by all other members of the research team until agreement was reached. The results were then presented to patient representatives and other members of the advisory group and discussed before being finalized.

## 3 Results

Interviews with participants identified three key themes related to patient involvement in providing feedback on their safety experiences: *cognitive‐cultural, structural‐procedural,* and *learning and change*.

### 3.1 Cognitive‐cultural

This theme represents how patients' conceptualizations of safety could influence their safety‐reporting behaviour. Within this theme, some participants discussed the importance of safety, whereas others felt it was not a concept relevant to them, and therefore not one they prioritized. The latter group had an assumption of safety as the “default position” of care delivery, and many felt that safety could not be isolated as a concept and instead had to be understood within the context of the complete health‐care experience.

#### 3.1.1 Perception that safety is important

Many participants reported that patient safety was a high priority for patients and staff, often drawing on their personal experiences of feeling safe. This can be seen in the extract below:

Yeah, well safety is a priority isn't it? Erm, well I always feel totally safe when I'm in there. I feel safe when I'm in hospital.

[P980]

The priority assigned to safety was further linked to patients' psychological safety, suggesting the importance of psychosocial safety, as demonstrated by Participant 1867; “Well I imagine [safety] is high on [staff's] list. It would help people to feel secure and get better you're not feeling stressed”. Psychosocial safety was also cited as important and relevant to patients' individual episodes of care, and to promoting longer‐term recovery and psychological well‐being. In particular, it was seen as important to reduce stress whilst in an unfamiliar hospital environment, as demonstrated by Participant 4300:

It was definitely emotional support that I needed [to feel safe] which is like just not me, so it's kind of completely out of character for me, so I didn't even know what was going on with my own emotions let alone what was going inside my body. So you know that was a tough time, so yeah that was, that was good care and you know I felt safe having them there, cos actually without them there I think I'd, well I don't know how I would have been.

[P4300]

#### 3.1.2 Safety is not the patient's priority

Other participants suggested that safety was not a priority for patients to think about. Many assumed that their safety was guaranteed during their stay in hospital and their transfer home, with trust placed in health‐care professionals within these clinical settings. For instance, Participant 104 stated that, “there's a question of safety to my mind, that doesn't come into it because I was in their hands…they were doing what they wanted, well they knew what they were doing.”

Whilst this perspective implied trust in health‐care staff and the health‐care system, it also suggested that safety was not something patients could offer a view on. Specifically, participants struggled distinguish the concept of safety from other aspects of care. Participant 104 discusses safety as a “side issue” alongside other aspects of care: “you don't go in there to be safe, you go in there to be mended […] Accommodation, transport, treatment, safety; that's what I'm trying to get at.” [P104]. Indeed, many patients took issue with the term “safety,” because they felt it was inadequate to capture their full experiences of care. Participant 3319 considered the word “safety” to be ambiguous within a context of having confidence (or trust) within health‐care staff. Conversely, for Participant 2494, safety was best understood as the receipt of satisfactory care and treatment.

I think this is quite ambiguous when you talk about safety I mean you perhaps intended to be ambiguous like that but I would have thought that confidence was perhaps a better word, do you have confidence in the nursing staff and in the doctors' confidence in the people that are attending to you rather than safety because as I say safety you kind of thing that you're in peril whereas you need to have confidence that are that you're putting your life in their hands really.

[P3319]

Interviewer Not the appropriate word? Ok that's interesting. Well what do you think would be a more appropriate word?

Participant Are you getting satisfactory care and treatment

[P2494]

When such attitudes are held, it is unlikely that patients would be inclined to respond to requests for their involvement in patient safety, for example by flagging up risks or completing feedback forms.

### 3.2 Structural‐procedural

This theme consists of two subthemes related to participants' attitudes towards the structures and processes of providing feedback. These were the *opportunity, means and ease* of providing feedback, and the *fear of reprisals* when doing so.

#### 3.2.1 Opportunity, means and ease

To provide feedback on their experiences of safety, participants noted that it was necessary for the process of doing so to be relatively easy and structured in a way to make it simple and straightforward to engage with. Specific examples related to surveys and feedback forms being brief, simple to answer and having a clear format. Participant 4300 commented that if a survey was too long, they would likely not complete it:

Smiley faces and sad faces and things like that, you know red faces, it looked simple it was easy it caught your eye it wasn't too wordy cos I think there's nothing worse than wordy surveys where you get half way through and you think you know what I can't be bothered.

[P4300]

Broader generalizations were also offered about how providing feedback can be an easy and trouble‐free process, with patients stating that they could see no reason not to provide it. Participant 2593 felt that patients should feel comfortable providing immediate feedback to staff:

I think patients should speak out more…. If patients are upset with how they are getting treated, they should be able to feel they can say something, there and then to whoever is looking after them.

[P2593]

Whilst some patients discussed the ease of providing feedback, others suggested ways in which the process was too difficult, and represented a barrier to providing feedback. Difficulties included the formatting, wording and an unclear purpose for requesting the feedback. The latter was linked to conceptualizations of safety, as can be seen in the below extract:

Well I suppose it's the job of the staff to look after you really, that's the way I would think of it. I mean, I shouldn't really have to complain about my own safety at all.

[P2450]

Others suggested that the process of giving feedback was generally too difficult, for themselves or other people. Reasons included tiredness, busyness and a general disinclination towards paperwork and surveys; particularly, once patients were removed from the care environment.

Participant I think once you've got yourself well you can't be bothered [to provide feedback].

Interviewer Yeah it's kind of behind you?

Participant Behind you, yeah.

[P3954]

#### 3.2.2 Fear of reprisals

For some patients, a fear of reprisals from staff was also a barrier to providing feedback. Even if the process was easy, some participants were dissuaded from providing feedback because they thought they might subsequently be treated poorly by clinical staff. Participant 2593 summarized this perspective when considering whether other patients would provide feedback on their safety experiences: “There are people in hospital that haven't been looked after and daren't say anything because they're frightened of reprisals.” Another patient told of an experience where they felt they had been blamed for providing feedback that resulted in a staff member losing their job:

Interviewer Ok, so you think, if you felt something wasn't safe and you said that, you would then get treated [differently]?

Participant Well I have been. When [I had] the problem, the epidural, I complained because obviously I was in a lot of pain. The Sister used to get a lift into work with the nurse that did it, she lost the job and so I got the blame, because she couldn't get a lift into work and everything. The treatment I got from her, on several visits and to stay at the hospital because I was always in the same ward. You just don't complain anymore.

[P1189]

Even if patients did not themselves fear such reprisals, some told stories of others who did. However, it should be noted that there were participants who explicitly stated that they did not believe such reprisals should be a cause of concern.

Participant I don't think so, I can't see that if they had a problem with certain staff, they would treat them any differently.

[P1867]

### 3.3 Learning and change

Regardless of what patients thought about the process of providing feedback, their views about the effectiveness of their feedback in promoting improvement were a crucial factor influencing whether they did so. Most of the participants felt that providing feedback to staff on the ward or to higher levels of governance would or could make a difference to safety in the future, as highlighted by Participants 980 and 3408:

Feedback is helpful in order to improve safety. If you did not give an opinion then they're not going to know what the patients want or what they didn't want

[P980]

You must give the right people feedback if there's any faults thrown up you can put them right

[P3408]

Those who expressed this view tended to be optimistic that staff wanted to do a good job, and that the right feedback would help them to do so and in turn create safer conditions, thus contributing to a capacity for quality improvement. Participant 4300 understood that patients and staff can have different perspectives, meaning that patient feedback on safety was necessary to avoid a staff assumption of satisfactory care. Furthermore, Participant 1867 asserted that there was a requirement for patients to play a role, even if “just” by providing feedback.

If you don't get feedback you don't know whether you're doing a good job or a bad job like in any walk of life. Like in my job you know if somebody doesn't tell me I'm doing a bad job then you think I'm doing a good job, because nobody goes out to purposely do a bad job, and you know nurses don't come onto the ward to purposely make you feel unsafe and to make you feel vulnerable and to give you a bad service. So they think they're doing good but they don't always see how you perceive it

[P4300]

I suppose [patients can make a difference to safety], if they have a feedback system. From work, they say everyone is legally responsible for safety. All the way from the patients to the top registrar you know, I'm assuming that they all see they have a part to play even if it's just feedback.

[P1867]

However, some interview participants were pessimistic about whether feedback would make a difference to safety. Some gave examples of times when they had made complaints with no clear outcomes; others spoke in more general terms, suggesting that feedback was ignored or dismissed as a nuisance. In both cases, feedback was perceived to have been ignored when the patients did not hear back from the staff members.

I've had lots of people in hospital and they tell me all this that's going off and you just think, nothing's getting any better and I've complained several times and put things in writing about different things, especially when my father was ill and you get nowhere, you get nowhere.

[P2593]

You tell the nurse [about problems] and the nurse thinks you're just being a bloody nuisance and she trots off and does her thing and forgets all about it. As far as I know, I mean she might, but I don't know because you don't get that feedback. There certainly is or was a lack of communication generally.

[P395]

## 4 Discussion

**Figure 1:**
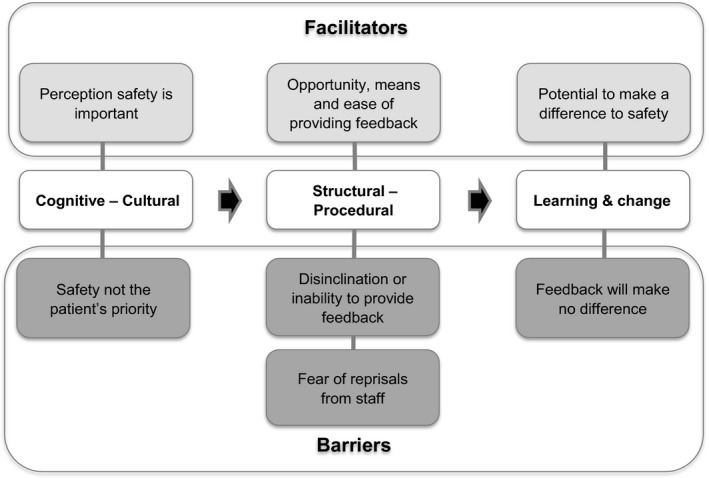
PReSaFe model of barriers and facilitators to **P**atients **Re**porting **Sa**fety **Fe**edback

This paper explored the barriers and facilitators to patients reporting their safety experiences, in terms of three key themes: *cognitive‐cultural*,* structural‐procedural*, and *learning and change*. Taken together, we argue that these themes form a staged model of barriers and facilitators (Figure 1), where each stage has different implications. Within this model, we hypothesize that each stage is a prerequisite for the next and that all are required for patients to report on their experiences. For example, a patient may understand the concept of safety (*cognitive‐cultural*), and there may be no *structural‐procedural* barriers in place, but if the patient does not think that feedback will lead to *learning and change*, they will be less likely to report their experiences.

The first component, *cognitive‐cultural*, relates to how patients conceptualized safety. Whilst most participants understood that safety was a priority, some felt that patient safety was not of relevance to patients. Where safety was deemed not relevant, patients reported that being safe was an assumed default position, or that safety was something that had to be understood within the context of the wider health‐care experience; thus, providing feedback on safety relating to discharge and care transfers is perceived as being of little utility. This finding is consistent with classic work by Hughes,^31^ who posited that the risk and responsibility for complex and risky activities can be transferred to a specialist rather than taken on by the individual themselves, if the specialist (ie the health‐care professional) was perceived as trustworthy and competent. This may account for the patients considering safety the “default” position. These “taken‐for‐granted” safety structures, as described by Rhodes et al.,^19^ make it difficult for patients to isolate safety from other aspects of their care experience. This difficulty in isolating particular elements of their experience was also reflected in participants' tendency to discuss their care experience as a whole, so that when asked specifically about their experience of care transfers, they discussed aspects of their hospital stay, apparently not viewing the transfer as a discrete part of their health‐care experience. Therefore, it may not be appropriate to ask patients to reflect on certain aspects of their experience, when they often consider the holistic experience, rather than a series of discrete stages.

Patients' conceptualizations of safety as identified in the *cognitive‐cultural* theme were different to standard academic understandings of safety, such as those proposed within Reason's model of safety,^3^ or the International Classification of Patient Safety.^32^, ^33^, ^34^ Whilst this is consistent with previous research,^13^, ^19^, ^35^, ^36^, ^37^, ^38^ it is important to highlight that this difference formed a major barrier to patients providing feedback on their safety experiences and raises the question of whether we should be using the term “safety” at all in materials aimed at patients. One approach to addressing this is to reconceptualize “safety” to incorporate patients' experiences. Another potentially complementary approach would be to develop models of health literacy to improve how patients understand the concept of safety. Health literacy work in patient safety has emphasized improving literacy among patients so that they are better able to participate in their health care,^39^ for example through improving patients' understanding of their medications.^40^ Such findings suggest that such improvements in literacy may also improve patients' readiness to report on safety incidents or experiences. However, there are concerns that current reporting structures may undermine patients' trust in clinicians.^10^ Therefore, it would be necessary to consider means of managing this appropriately, to ensure patients understand the value of reporting and do not perceive reporting on safety as complaining or as attribution of blame, but rather as the coconstruction of safety.

The second component of the model, *structural‐procedural*, was relevant to the process of providing feedback, with facilitators including the opportunity, means and ease of doing so. As suggested by the current study and previous literature,^16^, ^21^, ^22^ several barriers to patient involvement and reporting on safety exist. For example, Doherty et al.,^15^ identified that using existing clinician incident report tools to collect patient feedback resulted in a low number of responses, partly as a result of being a confusing process. Further structural‐procedural barriers identified in our study included disinclination or inability to provide feedback and fear of reprisals from staff; the latter resonates with a previous study, which identified patients' fear of being branded as difficult or as a nuisance as a barrier to reporting.^14^ An additional barrier that may result in patient disinclination to engage with reporting on safety includes lack of access to information about how to report issues. This again points to value in building health literacy among patients to address these barriers.

Recent work has shown that a positive environment for communication and mutual respect between health‐care professionals and patients can enable engagement and encourage patients to adopt an active role in their care.^41^ Therefore, providing an *explicit* opportunity for patients to provide feedback was considered a key enabler of patient reporting, which needs to be simple to understand to be effective. Strategies to support and reassure patients and to communicate the value of honest feedback may be required to ensure patients feel comfortable reporting without fear of reprisal.^42^

The final component, *learning and change*, represents the effectiveness of feedback. The perception that feedback has the potential to make a positive difference could facilitate patient reporting; conversely, the perception that feedback would not make any difference could inhibit patient reporting. Clear communication between health‐care professionals and patients may reassure patients that any feedback will be considered and will have an impact in terms of addressing concerns or issues. Previous research has highlighted the importance of avoiding a “black hole” of information reporting and effectively ensuring the safety feedback loop is closed,^43^ and this extends to patient complaints.^44^ It has been highlighted that learning and management systems are often de‐coupled from frontline practice, which can further intensify the views of patients and staff that safety reporting does not lead to improvement.^4^ Ensuring this feedback loop is closed and linking reporting mechanisms back to frontline staff and patients could help to address this issue and ensure that patient reporting is explicitly linked to quality and service improvement initiatives.^28^ This process would allow reported incidents and vulnerabilities to be addressed in a timely fashion and would promote trust in the reporting system by illustrating explicitly the positive effect that patient feedback can have on patient safety and quality improvement. Given that evidence indicates that patients differ from health‐care professionals in their perceptions and understanding of safety, patient feedback on safety experience can serve to act as an additional safety buffer against potential risks.^13^, ^28^, ^35^, ^36^, ^37^, ^38^ Furthermore, this approach is consistent with the NHS England's Sign up to Safety Campaign, which commits staff to listening, learning and responding to feedback from patients and staff by constantly measuring and monitoring the safety of services.^45^

A key strength of this paper is that it offers a model for understanding the barriers and facilitators to patients providing feedback on their safety, offering a testable framework for future research as well as considerations for those planning and designing patient feedback mechanisms. However, the research is not without its limitations. Some patients being discharged may not have been capable of taking part in an interview if there was not a family member or carer to assist them. Furthermore, due to the difficulty among participants in unpicking and reporting on discrete aspects of their care, it was challenging to ensure that participants focused on their experiences of safety within their care transfer during interviews. Given these findings, key learning points from this research are the need to reconsider the use of the word “safety” when asking patients to provide feedback on experiences, and to develop health literacy among patients such that they conceptualize it as an issue relevant to them, in which they can play an active and meaningful role.

## 5 Conclusion

Patient interviews offered important information about patients' receptiveness to reporting their safety experiences. To provide feedback on safety experiences, it was necessary for patients to conceptualize safety as something important and relevant to them. Both the ease of the process of providing feedback and the perceived effectiveness of that feedback could result in patients being more or less likely to provide feedback. The PReSaFe model proposed in this paper operationalizes barriers and facilitators to patients' reporting on their safety that we contend have relevance beyond the current work, by offering a testable framework for future work and potentially facilitating patient reporting on other experiences of care that are collected for quality improvement.

## Competing Interests

The authors have no competing interests to declare.
